# Differences in sex distribution, anatomic location and MR imaging appearance of pediatric compared to adult chordomas

**DOI:** 10.1186/s12880-016-0149-5

**Published:** 2016-09-08

**Authors:** Ronnie Sebro, Thomas DeLaney, Francis Hornicek, Joseph Schwab, Edwin Choy, G. Petur Nielsen, Daniel I. Rosenthal

**Affiliations:** 1 Musculoskeletal Radiology, Department of Radiology, Perelman School of Medicine, University of Pennsylvania, 3737 Market Street, Philadelphia, PA 19104 USA; 2 Department of Radiation Oncology, Massachusetts General Hospital and Harvard Medical School, 55 Fruit Street, Boston, MA 02114 USA; 3 The Harris Center for Chordoma Care, Massachusetts General Hospital, 55 Fruit Street, Yawkey 3B, Boston, MA 02114 USA; 4 Department of Orthopedic Surgery, Massachusetts General Hospital and Harvard Medical School, 55 Fruit Street, Boston, MA 02114 USA; 5 Department of Hematology/Oncology, Massachusetts General Hospital and Harvard Medical School, 55 Fruit Street, Boston, MA 02114 USA; 6 Department of Pathology, Massachusetts General Hospital and Harvard Medical School, 55 Fruit Street, Boston, MA 02114 USA; 7 Department of Radiology, Massachusetts General Hospital, Yawkey 6E, 55 Fruit Street, Boston, MA 02114 USA

**Keywords:** Chordoma, Pediatric, Adult, Chondroid, Dedifferentiated, Conventional, Spine, MRI

## Abstract

**Background:**

Chordomas are rare malignancies that primarily affect adults, but also rarely affect pediatric patients. We compared the imaging appearance, demographic and anatomic distributions of adult and pediatric chordomas in a large cohort.

**Methods:**

We performed a retrospective review of medical records of 220 subjects with histologically confirmed chordomas of the axial skeleton and pre-treatment magnetic resonance imaging studies. Age, sex, type of chordoma (conventional, chondroid or dedifferentiated), the anatomic location of the chordoma, as well as whether the lesion was primarily extra-osseous were recorded. Pediatric subjects were less than 21 years at the time of diagnosis. Binomial two-sample tests of proportions and Fisher’s exact tests were used to compare proportions between the pediatric and adult subjects.

**Results:**

Fifty six pediatric subjects (58.9 % female) and 164 adult subjects (42.1 % female) were identified. The proportion of female subjects with chordomas was significantly higher in the pediatric cohort compared to the adult cohort (*P* = 0.04). Most chordomas occur in Caucasians, however African-Americans were more represented in the pediatric cohort than in the adult cohort (*P* = 0.01). 69.6 % (39/56) of the pediatric chordomas involved the clivus/skull base and cervical spine compared to 29.3 % (48/164) of the adult chordomas (*P* = 1.99 × 10^−7^). Only 1.8 % (1/56) of the pediatric chordomas was in the sacrococcygeal region compared to 36.0 % (59/164) of the adult chordomas (*P* = 2.55 × 10^−8^). In cases where pre-treatment imaging was available, 93.8 % (16/17) of pediatric chordomas were predominantly extra-osseous compared to 76.7 % (46/60) of adult chordomas (*P* = 0.17).

**Conclusions:**

Pediatric chordomas more often affect females and occur most frequently at the craniocervical junction with decrease in incidence distally in the spine, whereas adult chordomas most frequently involve the craniocervical and sacrococcygeal regions.

## Background

Chordomas are rare, malignant tumors that bear a histological resemblance to cells of notochordal origin [1–3]. Chordomas have an incidence of approximately 1 in 1 million with approximately 300 new cases in the United States each year [4]. Chordomas predominantly arise in the clivus/skull base and/or spine [5, 6] with rare reports of extra-axial chordomas [7, 8]. Although chordomas predominantly affect middle-aged or elderly adults, they are also seen in the pediatric population (age < 21 years) [4, 9].

Pediatric chordomas are not well characterized in the literature due to the relative paucity of cases [4]. Anecdotal evidence suggests that pediatric chordomas tend to be predominantly extra-osseous with some involvement of the surface of the bone rather than an osseous tumor with a small soft tissue component and therefore greater than 50 % of the lesion tends to be extra-osseous.

We utilize a large cohort of subjects evaluated and/or treated for chordomas at the corresponding author’s institution to assess whether the sex and anatomic distribution of chordomas in pediatric subjects mirrors that seen in adult subjects, and evaluate the imaging appearance of chordomas to assess whether chordomas in pediatric subjects are more likely to be predominantly extra-osseous than chordomas in adult subjects.

## Methods

### Subjects

The study was approved by the Institutional Review Board (IRB) at the corresponding author’s institution and need for signed informed consent was waived.

This was a retrospective cohort study. Subjects had to have a pathologically proven diagnosis of chordoma for inclusion into the study. Histories were verified by searching a text database of all medical records and by review of all available imaging at our institution. Data was collected on subjects treated and/or evaluated at our institution between 01/01/1996 to 06/30/2014.

Data was collected on each subject’s age at diagnosis (based on age at the time of the initial pathology report), sex, self-reported race/ethnicity (Asian, African-American, Hispanic or Caucasian), type of chordoma (conventional, chondroid or dedifferentiated), anatomic location of the initial lesion (clivus/skull base, cervical spine, thoracic spine, lumbar spine, sacrococcygeal spine), and whether greater than 50 % of the pre-treated lesion was intra-osseous. Lesions that involved the clivus and skull base (above McGregor’s line) were considered clivus/skull base tumors. Lesions that involved the skull base and any component of the cervical spine (that is, lesions that extended inferior to McGregor’s line) were considered as cervical spine/skull base tumors.

### Imaging

Tumors were imaged using contrast-enhanced magnetic resonance imaging (MRI) performed on both 1.5 and 3 T systems. Measurements were obtained using the T2 imaging sequences. To assess whether a tumor was primarily intra-osseous or extra-osseous, the maximal intra-osseous component was identified in the axial plane and at that level, the maximal intra- and extra-osseous components were measured along the same plane. A lesion was considered primarily extra-osseous if there was more extra-osseous (>50 %) tumor at the level of the maximal intra-osseous tumor. All images were reviewed by a fellowship trained musculoskeletal radiologist.

### Statistical analyses

Binomial two sample tests for proportions were used to compare proportions between the pediatric and adult subjects. *χ*^2^ and Fisher’s exact tests were used to assess whether the anatomic location of the chordomas varied between pediatric subjects (subjects that were younger than 21 years of age) and adult subjects (subjects that were greater than or equal to 21 years of age at the time of diagnosis) and to test whether chordomas in pediatric subjects were more likely to be predominantly extra-osseous than chordomas in adult subjects.

*P*-values <0.05 were considered statistically significant. Statistical analyses were performed using R v2.9 software (https://www.r-project.org/).

## Results

We identified a total of 220 subjects (46.4 % female) with pathologically proven chordomas. Summary statistics were calculated for demographic variables and are shown in Table 1.

**Table 1:** Study sample demographics

	Pediatric subjects	Adult subjects	*P*-value
Age at diagnosis (mean, range)	10.0 (1–20)	55.8 (21–89)	<2.2 × 10^−16^
Sex (N, (% Male))	23 (41.1)	95 (57.9)	0.04
Race/ethnicity (N, (%))^a^			
Asian	0 (0)	4 (2.7)	0.61
Black/African-American	2 (5.3)	1 (0.7)	0.01
Hispanic	2 (5.3)	5 (3.3)	0.83
Caucasian	34 (89.5)	140 (93.3)	0.53
Type (N, (%))^b^			
Chondroid	10 (17.9)	34 (20.7)	0.79
Conventional	46 (82.1)	126 (76.8)	0.52
Dedifferentiated	0 (0.0)	4 (2.4)	0.55
Location (N, (%))^c^			
Clivus/skull base	35 (62.5)	47 (28.7)	5.0 × 10^−6^
Clivus/skull base and cervical	4 (7.1)	1 (0.6)	1.8 × 10^−2^
Cervical	10 (17.9)	25 (15.2)	0.74
Thoracic	3 (5.4)	13 (7.9)	0.77
Lumbar	1 (1.8)	18 (11.0)	0.07
Sacrococcygeal	1 (1.8)	59 (36.0)	2.4 × 10^−6^

^a^14 adult and 18 pediatric subjects declined to report race/ethnicity

^b^60 adult and 32 pediatric subjects’ pathology reports listed chordoma as final diagnosis. These were categorized as conventional chordomas based on discussion with the pathologist

^c^1 adult and 2 pediatric subjects did not have imaging confirmation of the anatomic location of their lesions

There were significantly more female subjects in the pediatric cohort (58.9 %) compared to the adult cohort (42.1 %) (*P* = 0.04). The majority of subjects in the combined cohorts were Caucasian (92.6 %), with relatively few Asian (2.1 %), African-American (1.6 %) or Hispanic (3.7 %) subjects. The ethnic/racial distribution of chordoma subjects in the pediatric cohort was significantly different from that in the adult cohort, with a higher proportion of pediatric subjects being Hispanic or African-American and a lower proportion of pediatric subjects being Asian or Caucasian compared to the adult subjects. We found that the proportion of African-Americans was statistically significantly higher in the pediatric cohort compared to the adult cohort (*P* = 0.01). The majority of the chordomas (78.2 %) were of the conventional subtype, which were approximately four times more numerous than the chondroid subtype in both cohorts. Dedifferentiated chordomas were not seen in the pediatric cohort, and only accounted for 2.4 % of the chordomas in the adult cohort. Approximately 20.7 % (34/164) and 76.8 % (126/164) of the adult chordomas, and 18.5 % (10/54) and 81.5 % (44/54) of the pediatric chordomas were of the chondroid and conventional subtypes respectively but there was no significant difference in frequency between adult and pediatric chordomas (*P* = 0.57).

**Fig. 1:**
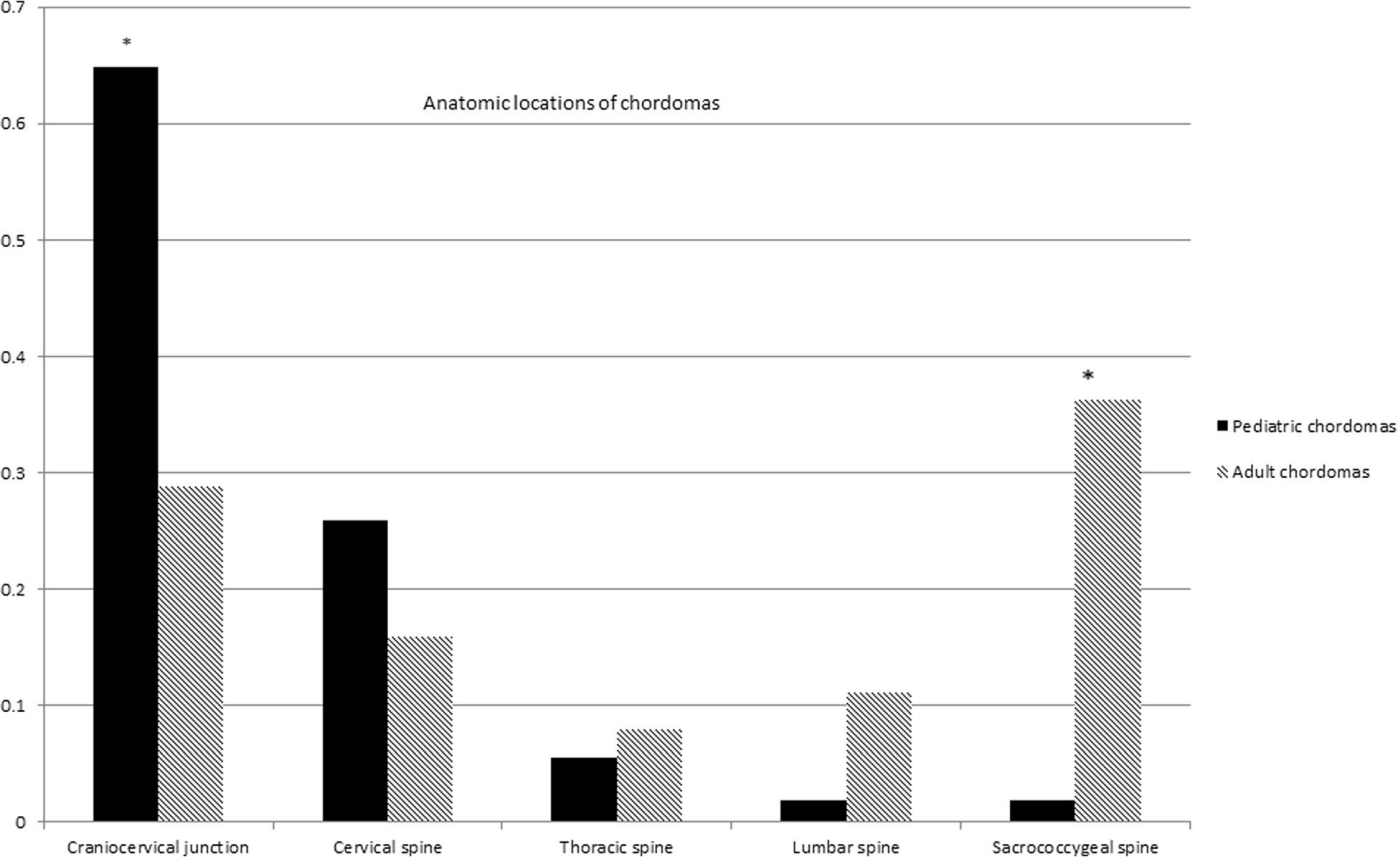
Anatomic location of chordomas. * *P*-value < 0.05

Analysis of the anatomic location of the primary chordoma lesions showed that in the pediatric cohort, the majority (72.2 %) of lesions were at the craniocervical junction (clivus and/or skull base and cervical spine) compared to only 29.6 % of the lesions in the adult cohort (*P* = 5.1 × 10^−8^) (Fig. 1). There was no significant difference in the anatomic location of the chondroid chordomas in the adult cohort compared to the pediatric cohort (*P* = 0.29), however there was a highly significant difference in the anatomic location of the conventional chordomas in the adult cohort compared to the pediatric cohort. There was a higher proportion of chordomas at the craniocervical junction in the pediatric cohort compared to the adult cohort (*P* = 1.24 × 10^−7^) and a higher proportion of chordomas as the sacrococcygeal region in the adult cohort compared to the pediatric cohort (*P* = 2.45 × 10^−7^) (Table 2).

**Table 2:** Comparison between anatomic location and type of chordoma^a^

	Pediatric				Adult			
Location	Ch	C*	D	Total*	Ch	C*	D	Total*
Clivus/skull base	9 [25.7] (90.0)	26 [74.3] (59.1)	0 [0.0] (0.0)	35 [100.0] (64.8)	21 [44.7] (61.8)	26 [55.3] (20.6)	0 [0.0] (0.0)	47 [100.0] (28.8)
Clivus/skull base and cervical spine	1 [25.0] (10.0)	3 [75.0] (6.9)	0 [0.0] (0.0)	4 [100.0] (7.4)	1 [100.0] (2.9)	0 [0.0] (0.0)	0 [0.0] (0.0)	1 [100.0] (0.6)
Cervical spine	0 [0.0] (0.0)	10 [100.0] (22.7)	0 [0.0] (0.0)	10 [100.0] (18.5)	2 [8.0] (5.9)	22 [88.0] (16.5)	1 [4.0] (33.3)	25 [100.0] (15.3)
Thoracic spine	0 [0.0] (0.0)	3 [100.0] (6.8)	0 [0.0] (0.0)	3 [100.0] (5.6)	1 [7.7] (2.9)	12 [92.3] (9.5)	0 [0.0] (0.0)	13 [100.0] (8.0)
Lumbar spine	0 [0.0] (0.0)	1 [100.0] (2.3)	0 [0.0] (0.0)	1 [100.0] (1.9)	2 [11.1] (5.9)	15 [83.3] (11.9)	1 [5.6] (33.3)	18 [100.0] (11.0)
Sacrococcygeal spine	0 [0.0] (0.0)	1 [100.0] (2.3)	0 [0.0] (0.0)	1 [100.0] (1.9)	7 [11.9] (20.6)	51 [86.4] (40.5)	1 [1.7] (33.3)	59 [100.0] (36.2)
Total	10 [18.5] (100.0)	44 [81.5] (100.0)	0 [0.0] (0.0)	54 [100.0] (100.0)	34 [20.9] (100.0)	126 [77.3] (100.0)	3 [1.8] (100.0)	163 [100.0] (100.0)

*Ch* chondroid

*C* conventional

*D* dedifferentiated

^a^1 adult with a dedifferentiated chordoma and 2 pediatric subjects did not have imaging confirmation of the anatomic location of their lesions

Numbers in square brackets [] represent the percentage of each chordoma histological subtype for each anatomic location i.e., row percentages

Numbers in round brackets () represent the percentage of chordomas in a given anatomic location for each histological subtype of chordoma, i.e., column percentages

*P-value <0.05 comparing pediatric to adult chordomas

35 % (76/220) of the subjects had pre-treatment imaging (prior to any radiation, surgery or chemotherapy) available for determining whether the chordoma was primarily intra-osseous or extra-osseous. Table 3 shows that most chordomas tend to have a substantial extra-osseous component, with 93.3 % of pediatric chordomas having a predominatly extra-osseous component compared to 76.7 % of adult chordomas. This difference was not statistically significant (*P* = 0.17).

**Table 3:** Comparison between pediatric and adult pre-treatment chordomas based on whether the lesion was predominantly intra-osseous or extra-osseous

Location	Pediatric subjects			Adult subjects		
	Predominantly intra-osseous	Predominantly extra-osseous*	Total*	Predominantly intra-osseous	Predominantly extra-osseous*	Total*
Clivus/skull base*	0 [0.0] (0.0)	11 [100.0] (73.3)	11 [100.0] (68.8)	3 [50.0] (21.4)	3 [50.0] (67.4)	6 [100.0] (10.0)
Clivus/skull base and cervical spine	0 [0.0] (0.0)	2 [100.0] (13.3)	2 [100.0] (12.5)	0 [0.0] (0.0)	1 [100.0] (2.2)	1 [100.0] (1.7)
Cervical spine	1 [100.0] (100.0)	0 [0.0] (0.0)	1 [100.0] (6.3)	0 [0.0] (0.0)	9 [100.0] (19.6)	9 [100.0] (15.0)
Thoracic spine*	0 [0.0] (0.0)	2 [100.0] (13.3)	2 [100.0] (12.5)	5 [100.0] (35.7)	0 [0.0] (0.0)	5 [100.0] (8.3)
Lumbar spine	0 [0.0] (0.0)	0 [0.0] (0.0)	0 [0.0] (0.0)	5 [71.4] (35.7)	2 [28.6] (4.3)	7 [100.0] (11.7)
Sacrococcygeal spine	0 [0.0] (0.0)	0 [0.0] (0.0)	0 [0.0] (0.0)	1 [3.1] (7.1)	31 [96.9] (67.4)	32 [100.0] (53.3)
Total	1 [6.3] (100.0)	15 [93.4] (100.0)	16 [100.0] (100.0)	14 [23.3] (100.0)	46 [76.7] (100.0)	60 [100.0] (100.0)

Numbers in square brackets [] represent the percentage of chordomas with a particular MR imaging appearance for each anatomic location i.e., row percentages

Numbers in round brackets () represent the percentage of lesions in a given anatomic location for chordomas with a particular MR imaging appearance, i.e., column percentages

*P-value < 0.05 comparing pediatric to adult chordomas

The proportion of predominantly extra-osseous chordomas was not statistically different by sex (*P* > 0.05) or subtype of chordoma (*P* > 0.05) in the combined cohort. However, sacrococcygeal chordomas and chordomas at the craniocervical junction were more likely to be predominantly extra-osseous compared to lesions in the thoracolumbar spine (*P* < 0.05).

## Discussion

Our work suggests that there are differences between pediatric and adult subjects with chordoma. Unlike adult subjects, pediatric subjects with chordomas are statistically more likely to be female. We also noted that there were slightly higher proportions of African-American/Black and Hispanic and slightly lower proportions of Caucasian and Asian subjects with pediatric chordomas compared to subjects with adult chordomas. It is unclear whether this change in ethnic/racial distribution is related to the referral pattern.

Our study shows that the distribution of the anatomic location of chordomas in pediatric subjects is different from that in adult subjects, with pediatric chordomas being primarily cranially located around the craniocervical junction decreasing in frequency towards to caudal spine whereas adult chordomas primarily occur at the most cranial and most caudal aspects of the spine. We do not know what accounts for the differences in chordoma anatomic location between the pediatric population and the adult population. Finally, we showed that the majority of the chordomas at the craniocervical junction and sacrococcygeal regions are predominantly extra-osseous compared to those in the thoracolumbar spine.

Our findings are in concert with previously published reports. Prior reports noted that there was a female predominance amongst pediatric subjects with chordomas [4, 10], and a male predominance amongst adult subjects [11] which is similar to our study. A report investigating the Surveillance, Epidemiology, and End Results Program (SEER) database of chordomas over approximately 30 years (1973–2003) showed that chordomas predominantly affect Caucasians with few African-American and Native American cases [11]. This is similar to our own results; however we also find that the self-reported race/ethnicity distribution changes in the pediatric population with chordomas.

It is sometimes stated that one of the diagnostic features of chordoma is that it involves the midline [12, 13]. It has been postulated that this is in some way related to residual notochord cells within the nucleus pulposus [12, 13]. The predilection for arising within fused segments (clivus, C2, sacrum) tends to support this idea, suggesting that notochordal remnant cells are somehow protected by the encasing bone as noted in our results. We noted that chordomas arising from the craniocervical junction and sacrococcygeal spine can be entirely or almost entirely extra-osseous or surface-based, and that this was less likely in lesions arising from the thoracic or lumbar spine.

It is unclear as to why the chordomas at the cranial and caudal aspects of the axial skeleton have substantial soft tissue components. It can be argued that sacrococcygeal tumors can reach a large size before becoming symptomatic given the potential large pre-sacral/pre-coccygeal space, and therefore large soft tissue masses remain clinically occult. However, there is substantially less potential pre-vertebral space in the craniocervical spine to accommodate a soft tissue mass and there are several adjacent neurovascular structures which should mean craniocervical chordomas should present with less of an extra-osseous component. Further research is required to understand whether this difference is clinically significant and whether thoracolumbar chordomas share similar genotyping and genomic profiles as well as response to therapy as craniocervical and sacrococcygeal chordomas.

Exophytic chordomas (chordomas with a predominant soft tissue component) at the cervical and craniocervical junction have been shown to be associated with increased risk of recurrence [14]. In addition, chordomas associated with the upper cervical spine and craniocervical junction have been shown to be independently associated with worse prognosis after adjusting for preoperative Frankel score, intralesional surgery, greater extent of invasion and revision surgery [15]. Our results show that pediatric chordomas are more likely to be in the upper cervical spine and at the craniocervical junction, and although these factors are associated with worse prognosis in the adult population, overall survival is longer and overall mortality is lower in pediatric patients [16], again highlighting a difference between pediatric and adult chordomas.

There are a few limitations to the analyses. The study is retrospective in nature and therefore subject to ascertainment bias. Our institution is a tertiary care center, and the majority of subjects were referred from other institutions for surgery, radiation therapy and/or second opinions, and the referral pattern may introduce a selection bias. We suspect that the self-reported ethnicity of the patients may be explained by selection/referral bias, and it is unclear whether this would influence sex and anatomic distribution. The race/ethnicity was not known for a large proportion of the pediatric subjects. Another limitation is that we did not distinguish between tumors that arise in bone and have a large soft tissue mass, from tumors that have an extra-osseous origin with some osseous involvement, because this differentiation can be very difficult even for musculoskeletal radiologists and sometimes contentious. However, we feel that our assessment presents the data in a format that can be reproduced by other researchers. Finally, because chordomas are rare, the small sample size limits the statistical power to detect other subtle differences that may exist between pediatric and adult subjects with chordomas.

## Conclusion

Chordomas in pediatric subjects are more likely to occur in females, the ethnic/racial distribution and anatomic distribution of chordomas differs between pediatric and adult subjects, with the majority of chordomas in pediatric patients occurring at the craniocervical junction, decreasing in incidence distally in the spine, whereas in adult subjects chordomas were more likely to occur in the sacrococcygeal region and craniocervical junctions, with very rare involvement of the thoracic and lumbar spine.

## Abbreviation

MRI, magnetic resonance imaging
